# The effect of foot posture on capacity to apply free moments to the ground: implications for fighting performance in great apes

**DOI:** 10.1242/bio.022640

**Published:** 2017-02-08

**Authors:** David R. Carrier, Christopher Cunningham

**Affiliations:** 1 Department of Biology, University of Utah, 257 S. 1400 E., Salt Lake City, UT 84112, USA; 2 Department of Biosciences, Swansea University, Swansea, Wales SA2 8PP, UK

**Keywords:** Plantigrade, Digitigrade, Free moment, Great apes, Male-male competition

## Abstract

In contrast to most other primates, great apes have feet in which the heel supports body weight during standing, walking and running. One possible advantage of this plantigrade foot posture is that it may enhance fighting performance by increasing the ability to apply free moments (i.e. force couples) to the ground. We tested this possibility by measuring performance of human subjects when performing from plantigrade and digitigrade (standing on the ball of the foot and toes) postures. We found that plantigrade posture substantially increased the capacity to apply free moments to the ground and to perform a variety of behaviors that are likely to be important to fighting performance in great apes. As predicted, performance in maximal effort lateral striking and pushing was strongly correlated with free moment magnitude. All else being equal, these results suggest species that can adopt plantigrade posture will be able to apply larger free moments to the ground than species restricted to digitigrade or unguligrade foot posture. Additionally, these results are consistent with the suggestion that selection for physical competition may have been one of the factors that led to the evolution of the derived plantigrade foot posture of great apes.

## Abstract (precis)

**Summary:** We tested the capacity of the heel-down foot posture of great apes to apply free moments to the ground because this could influence performance when fighting from a bipedal stance.

## INTRODUCTION

**Fig. 1.**
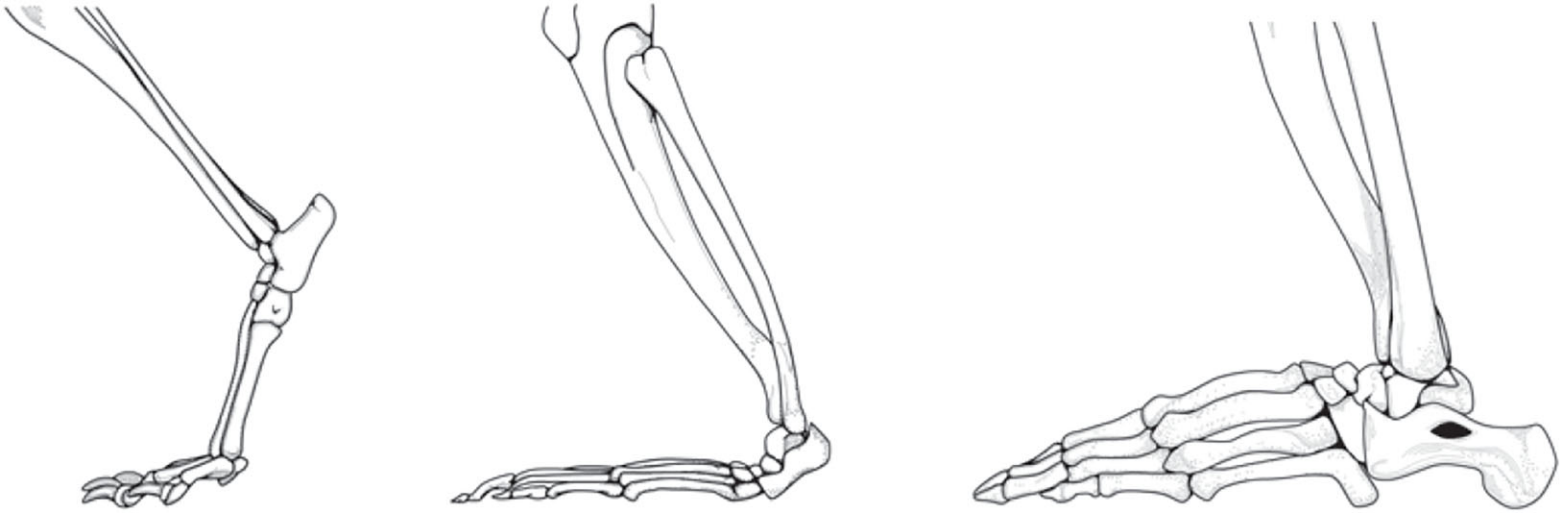
**Illustrations of the digitigrade locomotor foot posture that characterizes most therian mammals (e.g. dog; left), the semiplantigrade posture typical of most primates (e.g. monkey; center) and the plantigrade posture characteristic of all great apes (e.g. gorilla; right).** The illustration of the foot skeleton of the dog is modified from Hildebrand and Goslow (1998) and the skeletons of the gibbon and the gorilla are modified from Gebo (1992).

Plantigrade foot posture, in which the heel contributes to support of body weight during walking, running and standing, is a derived character of apes (Hominoidea) (Gebo, 1992; Schmitt and Larson, 1995; D'Août et al., 2002, 2004; Vereecke et al., 2003, 2005) (Fig. 1). Most primates walk, run, and stand with an elevated heel that does not contact with the substrate, the so-called semi-plantigrade posture. Walking gibbons (the lesser apes) exhibit an intermediate foot posture in which steps begin with contact by the metatarsal heads and this is followed by contact of the midfoot and the heel; a posture called ‘midfoot/heel plantigrade’ (Schmitt and Larson, 1995; Vereecke et al., 2005). However, the only study that has measured pressure under the feet of walking gibbons found that only one of four subjects actually loaded the heel during stepping (Vereecke et al., 2005). In contrast, weight support by the heel is consistently displayed by great apes (Hominidae). In great apes, both quadrupedal and bipedal steps begin with a heel strike (Gebo, 1992; Schmitt and Larson, 1995; D'Août et al., 2002, 2004; Vereecke et al., 2003) (Movie 1) and the heel contributes to support of body weight throughout the first half of the step (Schmitt and Larson, 1995; Vereecke et al., 2003). Thus, although some other primates have independently evolved plantigrade posture, for example spider monkeys (Schmitt and Larson, 1995), plantigrade posture is a derived trait of the apes and is most dramatically expressed in the great apes.

Several functional explanations for plantigrade foot posture have been proposed. Gebo (1992) suggested that the plantigrade feet of great apes evolved as a consequence of limits imposed on terrestrial locomotion by specialization for suspension climbing and brachiation. He argued that arms longer than the legs result in an inclined trunk when walking on the ground and the modified shoulder joint of great apes is less capable of supporting body weight in a quadrupedal stance. Thus, Gebo proposed that arboreal specialization produced a posterior shift of the body's center of mass and this led to contact of the heel with the ground during terrestrial walking and running. In contrast, Schmitt and Larson (1995) suggest that plantigrade foot posture evolved as a result of selection to reduce forelimb loading during quadupedal progression in species adapted for suspensory locomotion. They suggest that plantigrade posture is tied to greater hindlimb protraction at the beginning of a step and elevated activity of the hindlimb retractor muscles, which act to shift the center of mass posteriorly (Reynolds, 1985a,b). This active posterior shift of the center of mass increases reliance for weight support on the rear-hindfoot, including the heel. Thus, Schmitt and Larson (1995) suggest that the evolution of plantigrade foot posture is linked to a kinetic shift of the center of mass posteriorly to unload the forelimbs for improved reaching and grasping in an arboreal habitat (Jones, 1916; Clark, 1959; Reynolds, 1985a,b; Schmitt, 1998; Schmitt and Lemelin, 2002).

In humans, plantigrade foot posture also provides an energetic advantage during walking (Cunningham et al., 2010) and, to a lesser extent, during running at moderate speeds (Ogueta-Alday et al., 2014). Relative to most other species, humans are economical walkers (Sockol et al., 2007; Steudel-Numbers, 2003; Rubenson et al., 2007). Humans also differ from other studied species in that it costs substantially less to walk a given distance than to run the same distance (Rubenson et al., 2007; Farley and McMahon, 1992; McGeer, 1990; Margaria et al., 1963). The large energetic advantage during walking is due, in part, to a reduction in the loss of mechanical energy associated with the directional change of the trajectory of the center of mass during a walking step (Ruina et al., 2005; McGeer, 1990). Plantigrade feet reduce these directional changes and therefore decrease the loss of mechanical energy (Adamczyk et al., 2006; Cunningham et al., 2010). Humans are also known to have greater mechanical advantage at their limb joints during walking than during running (Biewener et al., 2004) and plantigrade foot posture plays an important role in this (Cunningham et al., 2010; Usherwood et al., 2012). Additionally, plantigrade posture increases effective limb length of walking humans (Webber and Raichlen, 2016). Thus, the structure of the human foot helps to explain why humans are economical walkers, but did plantigrade foot posture evolve in great apes to improve the economy of walking?

There are reasons to suspect that the heel-down posture of great apes did not evolve as a result of selection on economical locomotion; most species of extant apes travel relatively short distances. Daily distances traveled by orangutans, chimpanzees, bonobos and gorillas are 0.5–0.8 km (Bean, 1999), 3–10 km (Williams et al., 2002; Bean, 1999), 2.4 km (Williams et al., 2002; Bean, 1999) and 0.5–2.6 km (Doran and McNeilage, 2001; Bean, 1999), respectively. However, daily foraging distances of female and male human hunter-gatherers average 9.5 and 14.1 km, respectively (Marlowe, 2005). Second, the energy savings associated with high mechanical advantages at the limb joints of walking humans is a function of both plantigrade foot posture and of the highly erect limb posture of humans (Biewener et al., 2004; Cunningham et al., 2010; Usherwood et al., 2012). Great apes have plantigrade feet but walk with significantly flexed limb joints (Elftman, 1944; Sockol et al., 2007), further reducing the potential for energy saving from plantigrade foot posture. Thus, it seems unlikely that the evolution of plantigrade feet was driven by selection on locomotor economy in the last common ancestors of great apes.

Plantigrade feet may also improve performance when competing physically (i.e. fighting) with the forelimbs from a bipedal stance; a behavior that is common in all species of great apes (Livingstone, 1962; de Waal, 1982, 1986; Fossey, 1983; Goodall, 1986; Kano, 1992; Jablonski and Chaplin, 1993; Wrangham and Peterson, 1996; Furuichi, 1997; Thorpe et al., 2002) (Movies 1 and 2). Although moving rapidly from the balls of the feet is important in fighting, great apes (including humans) frequently strike and grapple with their heels in contact with the ground (Movie 1). By allowing the application of larger free moments to the ground, plantigrade foot posture may increase the application of force and energy when striking and grappling with an opponent. Striking or pushing on an opponent with the arm will result in a reaction force that tends to rotate the fighter's body. A competitor may be better able to strike and push with greater force and maintain position and balance if he or she can exert larger free moments on the ground with their feet. Free moments can be applied to the ground through two feet or through one foot that has two separated points of contact. Plantigrade posture may facilitate free moment production through two mechanisms: (1) maximizing the distance between the points of force application to the ground (i.e. moment arm), and (2) situating the rear ground contact behind the ankle joint. The rear ground contact in the semi-plantigrade feet of most primates is made through the anterior tarsal bones, the cuboid, entocuneiform and navicular (Vereecke et al., 2003). Although this allows a somewhat longer moment arm, between ground contact points of the anterior ankle and toes, the fact that the rear contact point is in front of the ankle means that it may act as a pivot point of the foot because it typically supports a greater percentage of body weight than the toes. For these reasons, we suspect that plantigrade foot posture will improve performance in behaviors important to success during fighting.

The ability to adopt plantigrade foot posture appears to be basal for mammals (Kielan-Jaworowska and Hurum, 2006). Plantigrade foot posture has been retained in some mammalian lineages (e.g. rodents), lost in some, and re-evolved in others (e.g. bears and great apes). Thus, the ability to apply free moments to the substrate during both locomotor and non-locomotor behaviors could be important to the evolution of foot posture in multiple mammalian lineages. Free moments have previously been studied in walking, running and turning (Holden and Cavanagh**,** 1991; Lee et al., 2001; Li et al., 2001; Jindrich et al., 2006; Umberger**,** 2008; Qiao et al., 2014). However, the role free moments play in non-locomotor behaviors and the extent to which foot posture influences free moment production have not been studied in any species. Although it is reasonable to hypothesize (1) that plantigrade feet may provide a larger moment arm for the application of free moments to the ground, and (2) that larger free moments applied to the ground could improve performance of some behaviors that are important when great apes fight, it is not clear that either of these predictions are true.

The suggestion that great apes are anatomically specialized for aggressive behavior has proven to be controversial. However, the mating systems of great ape species lead one to anticipate that specialization for aggressive behavior would have been important in their evolution. With the exception of bonobos, great apes are characterized by relatively high levels of male-male contest competition and intense physical aggression (Harcourt, 1978; Galdikas, 1985; Goodall, 1986; deWaal, 1986; Kano, 1992; Furuichi, 1997; Wrangham, 1999; Wrangham and Peterson, 1996; Puts, 2010, 2016; Wilson et al., 2014; Chatters, 2016; Daly, 2016; Rosenbaum et al., 2016; Hill et al., 2017). Additionally, as mentioned above, male great apes are unusual among primates because they routinely display and physically compete by striking and grappling with their forelimbs from a bipedal stance; a mode of fighting for which the proposed performance advantages of plantigrade feet could be particular relevant.

In this study we used human subjects to test the functional hypotheses that plantigrade foot posture (1) increases capacity to apply free moments to the ground and (2) thereby increases performance in grappling and striking behaviors that are employed by great apes when they fight. Ideally, we would like to test for performance differences between the basal semi-plantigrade posture of primates versus the derived plantigrade posture of great apes. This, however, is not possible in any extant species because of the immobility of the primate sub tarsal joints. Nevertheless, our experiments comparing digitigrade versus plantigrade posture in human subjects can falsify the hypotheses that plantigrade posture improves the ability to apply free moments to the ground and thereby increases performance in behaviors important for physical competition. Importantly, a finding that plantigrade foot does not increase performance in behaviors used by great apes when fighting would effectively falsify the evolutionary hypothesis that selection on performance during physical competition contributed to the evolution and retention of plantigrade foot posture in the lineage that gave rise to the great apes.

To test our primary prediction that plantigrade foot posture allows production of larger free moments, we asked human subjects to resist an increasing torsional moment applied to the long-axis of their body from both plantigrade and digitigrade foot postures. To determine the influence of foot posture on striking and grappling, we measured maximum effort performance and free moment production when subjects attempted relevant behaviors. We focused our analyses on forelimb striking and grappling behaviors that characterize fighting in chimpanzees and gorillas: lateral and downward strikes, lateral and forward pushes and rearward pulls (see Movies 1 and 2). We also measured performance in lateral pushing when subjects stood on a low coefficient of friction substrate that greatly limited the ability to apply free moments to the ground.

## RESULTS

### Maximum voluntary free moments from plantigrade and digitigrade posture

The maximum free moments exerted on the ground when subjects resisted vertically oriented long-axis torsion were larger when they resisted from plantigrade rather than digitigrade foot posture. The median peak free moments when the subjects resisted from one foot in digitigrade posture was 18.4 Nm (17.1–22.8 Nm, interquartile range) compared to 53.1 Nm (51.0–61.1 Nm) when they resisted from one foot in plantigrade posture (*P*-value=0.001, *N*=14). The median-peak free moments when the subjects resisted from two feet in digitgrade posture was 71.6 Nm (62.2–80.8 Nm) compared to 113.7 Nm (92.9–124.2 Nm) when they resisted from two feet in plantigrade posture (*P*-value=0.001, *N*=14). Thus, the median-maximum free moment produced by one foot in plantigrade posture was 165% (155–220%) greater than the free moment produced by one foot in digitigrade posture, and 58% (48–70%) greater when the subjects resisted with two feet in plantigrade posture than with two feet in digitigrade posture.

### Lateral strikes

The total energy delivered to the pendulum during maximum effort lateral strikes was greater when the subjects struck from plantigrade than digitigrade foot posture (Table 1; *N*=12). The median kinetic energy of one-foot strikes was 39.3% (24.8–48.6%, interquartile range) greater in plantigrade than in digitigrade posture and the median kinetic energy of two-feet strikes was 12.5% (8.8–20.0%) greater in plantigrade than in digitigrade posture. Additionally, both the impulse of the free moments and peak free moments were greater during strikes from plantigrade posture (Table 1). In the digitigrade trials, peak free moments were greater than (one-foot trials) or equivalent to (two-feet trials) the maximum voluntary free moments produced in the long-axis twisting trials (Table 2). Among the 12 subjects, kinetic energy was strongly correlated with free moment impulse for each of the four postures (Table 3). Additionally, kinetic energy and free moment impulse were strongly correlated when comparing mean values of the four postures within each subject (*N*=4). The median within subject Pearson correlation coefficient for the four postures of all of the subjects was 0.943 (0.916–0.989, *N*=12).

**Table 1.**
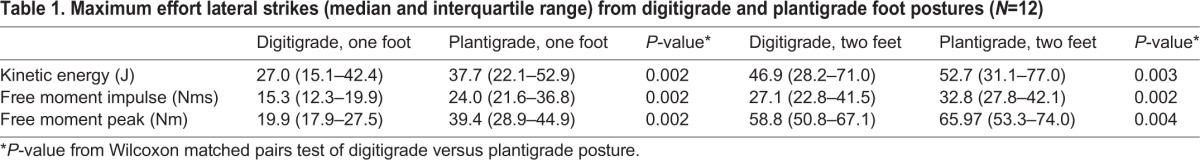
**Maximum effort lateral strikes (median and interquartile range) from digitigrade and plantigrade foot postures (*N*=12)**

**Table 2.**
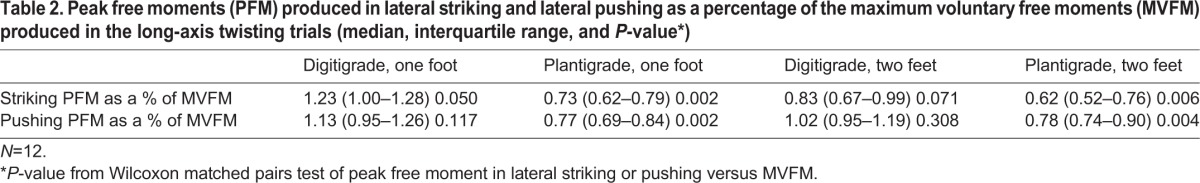
Peak free moments (PFM) produced in lateral striking and lateral pushing as a percentage of the maximum voluntary free moments (MVFM) produced in the long-axis twisting trials (median, interquartile range, and *P*-value*)

**Table 3.**
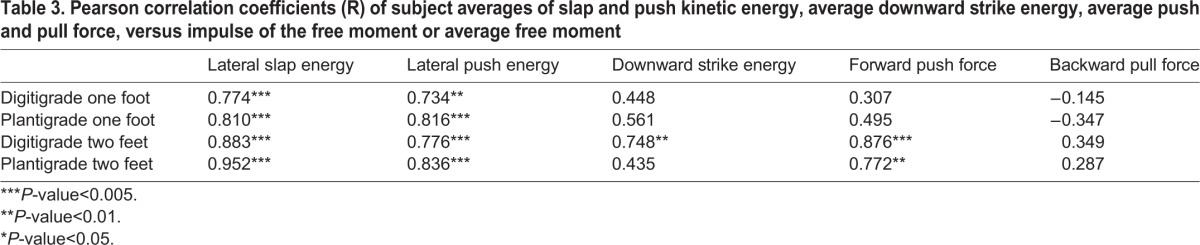
**Pearson correlation coefficients (R) of subject averages of slap and push kinetic energy, average downward strike energy, average push and pull force, versus impulse of the free moment or average free moment**

### Lateral pushes

The total energy delivered to the pendulum during maximum effort lateral pushes was greater from plantigrade than digitigrade foot posture (Table 4; *N*=12). The median kinetic energy of one-foot pushes was 23.8% (17.0–37.5%, interquartile range) greater in plantigrade than in digitigrade posture and the energy of two-feet pushes was 14.5% (8.0–25.7%) greater in plantigrade than in digitigrade posture. Both the impulse of the free moments and peak free moments were also greater during pushes from plantigrade posture (Table 4). In the digitigrade trials, peak free moments were equivalent to the maximum voluntary free moments produced in the long-axis twisting trials (Table 2). Among the 12 subjects, kinetic energy was strongly correlated with free moment impulse for each of the four postures (Table 3). Additionally, kinetic energy and free moment impulse were strongly correlated when comparing mean values of the four postures within each subject (*N*=4). The median within subject Pearson correlation coefficient for the four postures of all the subjects was 0.966 (0.949–0.983, *N*=12).

**Table 4.**
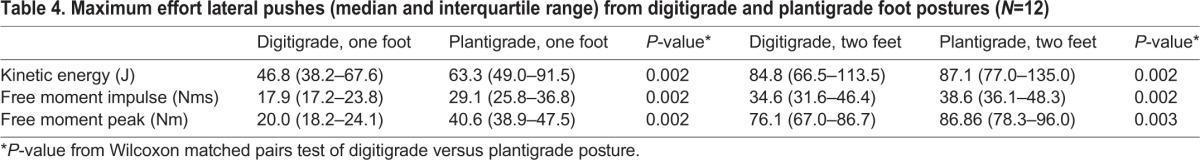
**Maximum effort lateral pushes (median and interquartile range) from digitigrade and plantigrade foot postures (*N*=12)**

### Lateral pushes when subjects stood on a substrate with a low coefficient of friction

To directly test the importance of free moments on performance in lateral pushing, we compared the energy generated in maximum effort lateral pushes when subjects stood on one foot on the normal sandpaper substrate versus a Teflon substrate that reduced the coefficient of friction between the subject's foot and ground by sixfold (Table 5; *N*=12). Relative to the low coefficient of friction substrate, the median kinetic energy of lateral pushes were 48.5% higher (34.7–101.3%, interquartile range) in digitigrade posture and 72.3% higher (53.4–98.9%) in plantigrade posture when the subjects stood on the high coefficient of friction substrate. Importantly, subjects were unable to maintain their forward facing orientation when pushing from the low coefficient of friction substrate; spinning about their vertical axis a median of 270^o^.

**Table 5.**
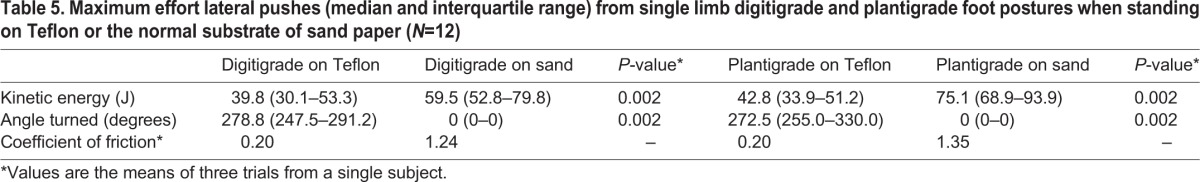
**Maximum effort lateral pushes (median and interquartile range) from single limb digitigrade and plantigrade foot postures when standing on Teflon or the normal substrate of sand paper (*N*=12)**

In the low coefficient of friction trials, we anticipated that the ability to apply energy to the transducer would be primarily limited by the subject's rotational inertia rather than by the free moments applied to the ground. If this were true, one would expect that there would be little or no difference in the energy applied to the transducer in the digitigrade and plantigrade postures when standing on the low coefficient of friction substrate. The median difference between plantigrade and digitigrade posture when the subjects stood on the low friction substrate was much lower, 9.2% (2.2–20.8%), than the median difference when they stood on high friction substrate, 21.9% (16.2–38.4%).

### Downward strikes

The total energy delivered to the pendulum during maximum effort downward strikes was slightly, but significantly, greater when the subjects struck from plantigrade than digitigrade foot posture (Table 6). The median kinetic energy of one-foot strikes was 5.7% (3.0–27.2%, interquartile range) greater in plantigrade than in digitigrade posture, and the energy of two-feet strikes was 5.5% (1.6–13.4%) greater in plantigrade than in digitigrade posture. Free moments produced during downward striking were of relatively small amplitude. Among the 11 subjects, the kinetic energy produced in downward strikes was significantly correlated with average free moment impulse in only one of the four postures (Table 3).

**Table 6.**
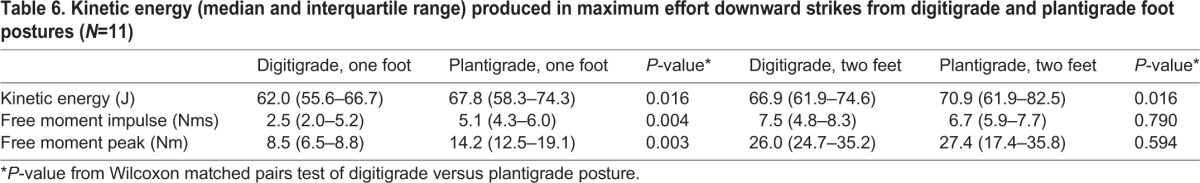
**Kinetic energy (median and interquartile range) produced in maximum effort downward strikes from digitigrade and plantigrade foot postures (*N*=11)**

### Static forward pushes

The average force applied during maximum voluntary effort static forward pushes was greater when the subjects pushed from plantigrade than digitigrade foot posture (Table 7; *N*=11). The median average force of one-foot pushes was 70.4% greater (43.4–83.6%, interquartile range) in plantigrade than in digitigrade posture and the median average force of two feet pushes was 37.4% greater (23.1–51.6%) in plantigrade than in digitigrade posture. The median free moments were also greater during pushes from plantigrade posture (Table 7). Among the 11 subjects, average force was strongly correlated with average free moment for the two-foot trials, but not for the one-foot trials (Table 3). Additionally, average pushing force and average free moment were strongly correlated when comparing mean values of the four postures within each subject (*N*=4). The median within subject Pearson correlation coefficient for the four postures of all the subjects was 0.962 (0.867–0.996, *N*=11).

**Table 7.**
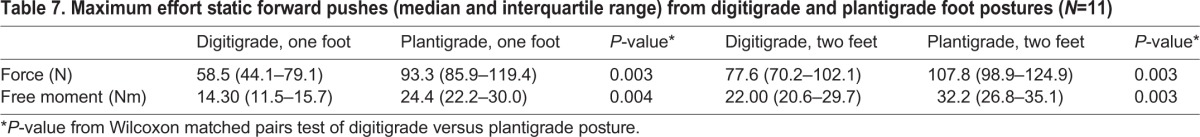
**Maximum effort static forward pushes (median and interquartile range) from digitigrade and plantigrade foot postures (*N*=11)**

### Static rearward pulls

The average force applied during maximum effort static rearward pulls was greater when the subjects pulled from plantigrade, rather than digitigrade, foot posture (Table 8; *N*=11). The median average force of one-foot pulls was 26.6% greater (19.4–32.5%, interquartile range) in plantigrade than in digitigrade posture, and the median average force of two feet pulls was 13.4% greater (8.7–32.3%) in plantigrade than in digitigrade posture. Although not significantly different, the average free moments tended to be greater during pulls from plantigrade than digitigrade posture (Table 8). Among the 11 subjects, average pulling force was not correlated with average free moment (Table 3). However, within each subject, average force and average free moment tended to be correlated across the four postures (*N*=4). The median within subject Pearson correlation coefficients for the four postures of all the subjects was 0.830 (0.601-0.967, *N*=11).

**Table 8.**
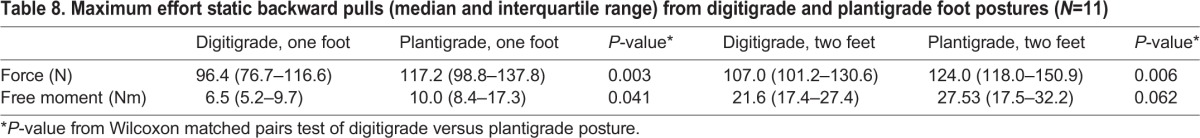
**Maximum effort static backward pulls (median and interquartile range) from digitigrade and plantigrade foot postures (*N*=11)**

### The impact of postural instability on performance when standing in digitigrade posture

To address the influence of postural instability (i.e. balance) on performance during digitigrade trials compared with plantigrade trials, we compared performance of subjects when they completed lateral pushing trials with and without stabilizing support from their left (non-pushing) hand (Table 9; *N*=8). Stabilization was provided by resting the side of the hand against a stable vertical support. Hand support greatly increased postural control, increasing the time that subjects could stand digitigrade on one foot with their eyes closed from a median of 3.6 s to greater than 30 s in all subjects. Although the hand support did tend to increase performance in lateral pushing in both the single-foot digitigrade and plantigrade postures, the increase was not statistically significant (*P*>0.025) in either case. Most importantly, the increase in pushing performance when subjects switched from single-foot digitigrade to plantigrade posture was not significantly different between the no-support [23.1% (17.2–50.9%)] and hand-support [18.2% (13.5–37.3%)] trials (*P*=0.575).

**Table 9.**
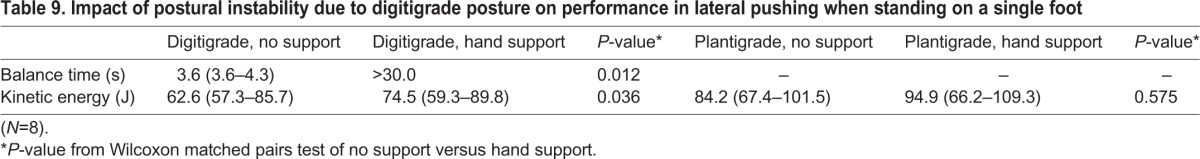
**Impact of postural instability due to digitigrade posture on performance in lateral pushing when standing on a single foot**

## DISCUSSION

### Effect of foot posture on maximum effort free moment production

The primary finding of this study is that plantigrade foot posture increases the ability to apply free moments to the ground. In the resistance to long-axis torsion trials, maximum free moments produced in plantigrade posture were 165% greater than those produced in digitigrade posture when the subjects resisted from one foot, and 58% greater when they resisted from two feet. We suspect that contact of the heel with the ground facilitates free moment production by (1) maximizing the distance between the points of force application to the ground (i.e. moment arm), and (2) situating the rear ground contact behind the ankle joint, thereby increasing the capacity of the rear contact point to apply a force couple rather than act as a pivot point. Nevertheless, these results suggest that, all else being equal, species that are able to adopt plantigrade foot posture, such as bears, badgers, wolverines, many rodents, spider monkeys, and great apes, should be able to apply greater free moments to the ground than species that are restricted to digitigrade or unguligrade foot posture.

The increase in free moments produced in the one-foot trials when subjects switched from digitigrade to plantigrade posture was likely due to the increase in length of the moment arm associated with the distance between the ball of the foot and toes versus the distance between the heel and the toes. However, moment arm length cannot explain why plantigrade posture increased performance in a two-foot stance. If the relevant moment arm was the distance between the two feet, the maximum free moment should not be affected by the switch from plantigrade to digitigrade foot posture. The fact that maximum free moment was greater in the two-foot plantigrade trials, suggests that contributing force couples are applied by one or both of the plantigrade feet as well as by the two feet working together.

Our visual monitoring of the subjects during maximum resistance to long-axis torsion provided some indication of what limited performance in these trials. During the single-foot trials, recording was terminated when the subject's foot began to spin on the force plate, indicating that friction was limiting. This was true for both digitigrade and plantigrade posture. In contrast, during the two-foot trials the feet rarely slipped and the subjects appeared to be limited by an unidentified component of leg or trunk strength.

### Effect of foot posture on performance in striking and shoving

Relative to digitigrade foot posture, plantigrade posture improved performance in lateral striking, lateral pushing, downward striking, forward pushing and rearward pulling. In the digitigrade trials, peak free moments produced during lateral striking and pushing were greater than or equivalent to the maximum voluntary free moments produced in the long-axis twisting trials. Importantly, strong correlations were found between performance and free moments applied to the substrate in lateral striking, and lateral and forward pushing. Additionally, when the capacity to apply free moments to the ground was limited by having subjects stand on a substrate with a low coefficient of friction, performance in lateral pushing was dramatically reduced and subjects spun about their vertical axis. These results (1) indicate that factors that limit the ability to produce free moments, such as foot posture and substrate friction, can limit performance in behaviors that involve the application of long-axis rotational torques to the body; and (2) suggest that the capacity of a plantigrade foot to exert free moments on the ground does provide an advantage in some behaviors that are likely to be important to success in fighting.

To address the possibility that the observed differences in performance between digitigrade and plantigrade foot posture were a result of decreased stability when standing in digitigrade posture, we ran trials in which stability in digitigrade stance was increased through support with the non-pushing hand. A series of studies on postural control have shown that light touch with the fingers or hand on a support provides an effective orientation reference that improves balance control in an upright stance (Jeka, 1997). Thus, the finding that the increase in lateral pushing performance when subjects switched from single-foot digitigrade to plantigrade posture was not significantly different between the normal no-support and the hand-support trials, indicates that decreased stability associated with digitigrade posture cannot explain the observed performance differences in digitigrade versus plantigrade performance.

### Evolutionary significance of plantigrade feet in great apes

Although there are a variety of possible explanations for the evolution of plantigrade foot posture in Hominidae, the findings that plantigrade foot posture does have functional consequences for locomotor energetics (Cunningham et al., 2010; Usherwood et al., 2012; Ogueta-Alday et al., 2014; Webber and Raichlen, 2016), as well as for striking and grappling behaviors (this study), provide evidence against the evolution of plantigrade foot posture due to stochastic evolutionary process, such as genetic drift. Similarly, although it is possible that the unusual foot posture of the great apes is the result of a developmental constraint, there is no current evidence to support this non-mutually exclusive possibility. Within the explanatory realms of natural and sexual selection, there are many untested behaviors for which plantigrade posture may provide a performance advantage. However, as described above, there is evidence to suggest that selection for suspension locomotion in an arboreal habitat (Gebo, 1992; Schmitt and Larson, 1995) as well as selection for more economical locomotion could have played a role in the evolution of plantigrade foot posture of great apes. Additionally, the results of this study lead us to propose that selection on fighting performance must be added to this list of possible functional explanations for the evolution of plantigrade foot posture in Hominidae.

In this context, plantigrade foot posture may be part of a suite of adaptations for intraspecific fighting in Hominidae. The pronounced sexual dimorphism in body size (Plavcan, 2001, 2003, 2012; Zihlman and McFarland, 2000, Zihlman and Bolter, 2015, reviewed by Carrier and Morgan, 2015) and upper body strength (Zihlman and McFarland, 2000), as well as the relatively short legs of great apes (Carrier, 2007) are consistent with specialization for fighting performance. Within the bipedal apes, the hominins, additional characters that have been suggested to be consistent with specialization for fighting include habitual bipedalism (Darwin, 1871; Livingstone, 1962; Wescott, 1963; Jablonski and Chaplin, 1993; Carrier, 2011), hand proportions that protect the hand when it is used as a fist to strike (Morgan and Carrier, 2013; Horns et al., 2015), increased robusticity of the face (Puts, 2010; Carrier and Morgan, 2015), and sexual dimorphism in upper body strength (McHenry, 1986, 1991, 1996; Bohannon, 1997; Abe et al., 2003; Lassek and Gaulin, 2009; Vasavada et al., 2001), stature (Carrier, 2011; Stulp et al., 2015), facial robusticity and shape (Sell et al., 2009; Puts, 2010; Carrier and Morgan, 2015), and voice (Puts et al., 2006; Puts, 2010; Sell et al., 2010; Hill et al., 2017). Additionally, in modern humans, the size of the muscles of the back and leg appear to be more related to the demands of explosive behaviors that are likely to be important in physical competition than the demands of high speed sprinting or sustained endurance running (Carrier et al., 2015). Thus, many traits within Hominidae, including several of those most diagnostic of both Hominidae and hominins, may be partially a consequence of sexual selection on fighting ability in males.

## MATERIALS AND METHODS

We tested the ability of human subjects to apply free moments to the ground when they performed from two different foot postures: plantigrade posture in which the heel was in contact with the ground and helped support the body, and digitigrade posture in which the heel was elevated such that the body was supported by the ball of the foot and toes (Fig. 1). In addition, we tested the effect of foot posture on performance in several behaviors that are likely to be relevant to success during physical competition lateral striking and pushing, downward striking, and forward pushing and rearward pulling. All trials were completed barefoot, except in one case in which subjects wore a sock on their right foot, described below. Nineteen healthy males served as subjects in one or more of the experiments. The sample size for individual tests varied from 8–14 subjects. Subjects gave informed written consent. All procedures were approved by the University of Utah Internal Review Board (IRB_00073326). Informed consent was obtained from all participants, and all aspects of the study complied with the Declaration of Helsinki. Data collected and analyzed in this study are available at the Dryad Digital Repository (Carrier and Cunningham, 2017).

### Rationale for the fighting behaviors studied

Although the social contexts in which fights occur and the injuries that result are often described in the behavioral literature, the actual fighting behaviors used by great apes are rarely mentioned. A few descriptions of how apes fight, however, do exist. Detailed, eyewitness accounts of lethal fighting in chimpanzees are provided by Goodall (1986). In these fights, groups of three to six adult males attacked isolated individuals, usually males, from an adjacent community. The attacks began by grappling and pulling the victim to the ground, in some cases from out of a tree the victim had attempted to flee into. The victim was held, pinned to the ground by one member of the group while other members attacked by biting, hitting with hands, and kicking and stomping on. The victims were often dragged for distances on the ground, lifted and slammed back to the ground, and attempts were made to break arms and legs by twisting. In these lethal attacks, a bipedal stance was used as a base for grappling, striking with the forelimbs, as well as lifting, dragging, and twisting the victim. Among bonobos, physical aggression includes pulling, slapping, hitting, shoving aside, pinning down and biting (Hohmann and Fruth, 2003). Fights between male orangutans are reported to involve grappling and biting (Galdikas, 1985). We are not aware of published descriptions of how gorillas fight. However, male gorillas housed together in zoos grapple and hit each other with laterally and downward directed strikes (Movies 1 and 2). Thus, to access the impact of foot posture on behaviors that appear to be important in great ape fighting, we focused on lateral striking and pushing, downward striking, forward pushing and rearward pulling. We did not include forward punching with a closed fist in this study because it is a derived behavior of hominins (Morgan and Carrier, 2013; Carrier and Morgan, 2015), and therefore evolved long after the evolution of plantigrade feet.

### Analysis of free moments

Free moments generated by the subjects during maximal effort trials were measured as a force couple by the horizontal sensors of a Kistler 9281B SN force plate (Amherst, NY, USA). Forces applied to the force plate were sampled at 400 Hz with a National Instruments analog-to-digital converter (Austin, TX, USA) and stored on a Macintosh computer. To provide a suitable level of friction between the subject's feet and the force plate, the surface of the force plate was covered with 120 CAMI grit sandpaper. Force outputs from the horizontal sensors were summed to yield the net horizontal force. The appropriate fraction of net horizontal force (determined by the proximity of the center of pressure to the sensors) was subtracted from the outputs of two parallel sensors to remove translational components, yielding equal and opposite forces with parallel lines of action (a force couple). One of these forces was then multiplied by the distance between the sensors to yield a moment. This procedure was carried out for both components of the horizontal force, and their moments were summed to give the free moment (Holden and Cavanagh, 1991).

### Analysis of maximum free moments generated while resisting vertical torsional loading

To measure the maximum free moments subjects could apply to the ground from the different foot postures, we asked subjects to resist a vertical twisting torque applied to their trunk as they stood barefoot on the force plate. The subjects held a steel post, 1.83 m long, across their mid-back with the crooks of their arms. Two experimenters, holding the ends of the post, applied a clockwise force couple directed along the long axis of the body. The experimenters gradually increased the magnitude of the force couple until the subjects' feet began to slide on the surface of the force plate or the strength of the subject was exceeded. The distance the subjects' feet slid on the sandpaper was minimized so that they experienced little or no discomfort. Trials in which subjects lost their balance were not saved. Two trials were recorded for each of four postures: one-foot (right foot) digitigrade and plantigrade posture and two-feet digitigrade and plantigrade posture. The sequence of the foot postures was varied randomly between subjects. Fourteen healthy male subjects participated in this experiment [body mass 78.5, 71.7–85.0 kg (median and interquartile range); age 28.5, 25.5–38.5 years].

### Analysis of lateral strikes and pushes

**Fig. 2.**
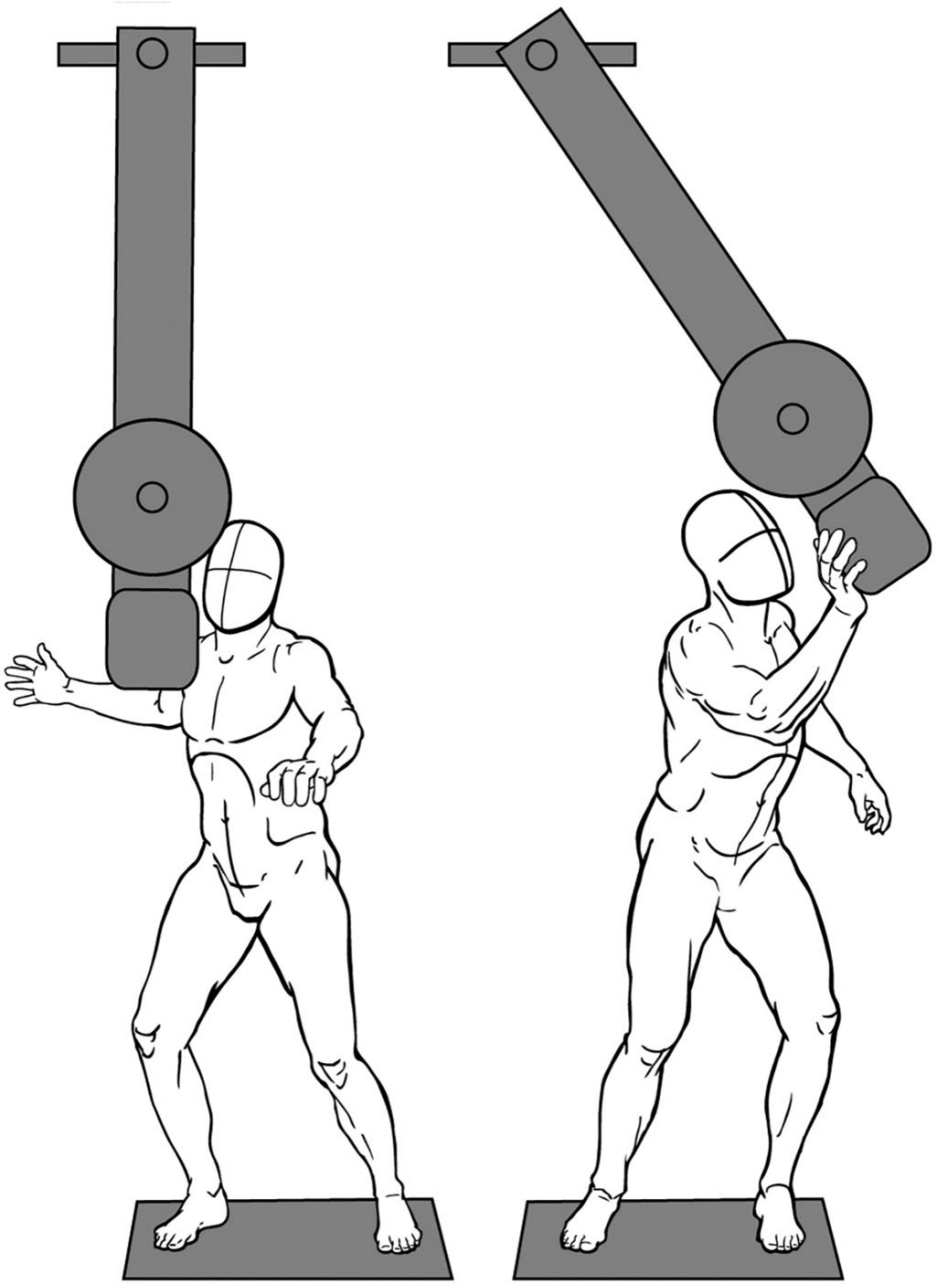
**Illustrations of the pendulum transducer used to measure the energy imparted during maximum effort laterally directed strikes and pushes.** (A) Prior to strike and (B) mid strike. Subjects stood on a force plate to allow recording of the free moments applied by the feet to the ground.

We measured performance in lateral striking and pushing with an instrumented pendulum (Fig. 2). The energy delivered to the pendulum by the subject was determined by calculating the change in kinetic energy, which is one half the rotational inertia of the pendulum times the maximum angular velocity squared. Angular velocity of the pendulum was measured with a gyroscope (Yaw Rate Gyroscope v1.0, Vex Robotics, Greenville, TX, USA). The rotational inertia of the pendulum was calculated to be 20.97 kg m^2^. Subjects stood on a force plate during striking and pushing to allow monitoring of free moments. Three trials were recorded for each of the four postures: one-foot (right foot, i.e. ipsilateral) digitigrade and plantigrade posture and two-feet digitigrade and plantigrade posture. The sequence of the foot postures was varied randomly between subjects. Twelve healthy male subjects participated in this experiment [body mass 77.5, 70.3–83.5 kg (median and interquartile range); age 27.0, 25–34 years].

### Testing the importance of free moments by reducing substrate coefficient of friction

As an additional test of the role of free moment production in behaviors that involve reaction forces that act to spin the body about its vertical axis, we compared performance in lateral pushing when subjects stood on the normal sandpaper to when they stood on a sheet of Teflon that greatly reduced the coefficient of friction between the substrate and the subject's foot. In the pushing while standing on Teflon trials, the ability to apply energy to the transducer was presumably limited by the subject's rotational inertia rather than the free moments applied to the ground. Subjects stood on their right foot in digitigrade and plantigrade posture and completed maximum effort lateral pushes against the instrumented pendulum, as described above. For the trials done while standing on Teflon, the subjects wore a sock to further reduce the coefficient of friction. In the Teflon trials, we recorded the angle the subject's body spun by noting the direction the subject's trunk faced once they stopped spinning with angles marked on the ground at 30-degree intervals. Twelve healthy male subjects participated in this experiment [body mass 78.1 kg, 73.2–83.2 kg (median and interquartile range); age 26.5, 24–30 years].

In one subject, we measured the static coefficient of friction of a barefoot standing on sandpaper and a sock-clad foot standing on Teflon, by pulling the subject across the forceplate with a Nylon strap wrapped around the front of the subject's ankle. The distance the subject's foot slid on the sandpaper was minimized so that he experienced only minor discomfort. The static coefficient was calculated as the friction force (i.e. horizontal) divided by the normal force (i.e. vertical). Average values are reported from three trials from digitigrade and plantigrade posture for both substrates.

### Analysis of downward strikes

We measured performance in downward striking with the instrumented pendulum used in a previous study (Carrier, 2011). As with the lateral striking and pushing trials, the energy imparted to the pendulum by the subject was determined by calculating the change in kinetic energy. Angular velocity of the pendulum was measured with a gyroscope (Yaw Rate Gyroscope v1.0, Vex Robotics, Greenville, TX, USA). For safety, the rotational inertia of this pendulum was substantially lower than that of the pendulum used to monitor lateral striking and pushing and was calculated to be 1.98 kg m^2^. The sequence of the foot postures was varied randomly between subjects. Eleven healthy male subjects participated in these experiments [body mass 82.1, 76.4–90.8 kg; age 27, 25–31 years (medians and interquartile ranges)].

### Analysis of static forward pushes and rearward pulls

To quantify performance when the subjects pushed and pulled from plantigrade and digitigrade foot posture, the subjects stood on the force plate as they pushed or pulled with one arm in the parasagittal direction, against a non-extensible line attached to two S-beam load cells arranged in parallel (LCR-50, Omega, Stamford, CT, USA). Subjects were instructed to gradually increase the applied force to maximum effort and hold it for 2 s. We calculated the average force applied to the transducers and the average free moment applied to the force plate during this period. In the one-foot trials, subjects stood on the foot ipsilateral to the arm they were pushing or pulling with. Three trials were recorded for each of the four postures: one-foot (right foot) digitigrade and plantigrade posture and two-feet digitigrade and plantigrade posture. The sequence of the foot postures was varied randomly between subjects. Eleven healthy male subjects participated in these experiments [body mass 82.1, 76.4–90.8 kg; age 27, 25–31 years (medians and interquartile ranges)].

### Testing the impact of digitigrade instability on performance

Because greater postural instability in digitigrade posture may have impacted comparisons of performance in digitigrade to plantigrade posture, we compared performance in maximal effort lateral pushing trials when subjects used their left hand to stabilize themselves to trials which did not use hand stabilization. These trials were all done when the subjects stood on their right foot and were completed as described above. In the stabilization trials, subjects braced the ulnar side of their left hand against a fixed vertical surface immediately to their left, at the height of their mid-abdomen. By resting only the side of their hand on the vertical support, subjects were not able to apply horizontally directed pulling forces on the support, which could have increased the torque applied to the pendulum. Subjects were instructed to brace with their hand, being careful not to lean against the support. To access whether or not this hand bracing increased balance stability, we measured the time subjects could retain their balance when standing on one foot with digitigrade posture with their eye closed, with and without hand bracing. Eight healthy male subjects participated in these experiments [body mass 80.8, 73.2–83.5 kg; age 26.5, 25.0–34.0 years (medians and interquartile ranges)].

### Statistical analysis

Because we suspect outlier trials are meaningful, analysis was done on mean values of the individual subject trials. Median and 25% and 75% quartile values of the means of the subject were calculated and are reported. To test whether performance in digitigrade and plantigrade posture was different, we used the Wilcoxon matched pairs test to compare subject mean values. We assumed the results were significantly different when the *P*-value was less than 0.05. In cases in which we tested for differences between foot posture in both one-foot and two-feet trials we adopted a simple Bonferroni correction and assumed the results were significantly different when the *P*-value was less than 0.025. All tests were two-tailed. To determine if performance in striking, pushing, pulling was correlated with the free moment applied to the ground we calculated the Pearson correlation coefficients (R) of subject means of slap and push kinetic energy, mean push and pull force, or turn angle, versus subject mean impulse of the free moment or peak free moment.
